# Feedback literacy in EFL listening: structural associations and reported action strategies among Chinese university students and instructors

**DOI:** 10.3389/fpsyg.2026.1855025

**Published:** 2026-08-05

**Authors:** Yanfang Zhang, Jinyan Huang, Ying Liu, Jai Kumar

**Affiliations:** 1 Beijing Union University, Beijing, China; 2 Jiangsu University, Zhenjiang, China; 3 The Chinese University of Hong Kong, Hong Kong, Hong Kong SAR, China; 4 Xiangnan University, Chenzhou, China

**Keywords:** appreciating feedback, Chinese university students, EFL listening, evaluative judgment, feedback literacy, managing affect, mixed methods, taking action

## Abstract

Feedback literacy describes the understandings, capacities, and dispositions through which learners recognize the value of feedback, make evaluative judgments, manage emotional responses, and take action. This mixed-methods study examined these four interrelated features in English-as-a-foreign-language (EFL) listening among 1,901 Chinese non-English-major undergraduates and explored action strategies reported by students and 18 college English instructors. The student questionnaire comprised 20 researcher-developed items contextualized to EFL listening; 19 indicators were retained after one taking-action item showed a loading below 0.50. Partial least squares structural equation modeling was used to examine theory-informed associations, and student open-ended responses and instructor interviews were analyzed through qualitative content analysis. Internal-consistency and convergent-validity indices were acceptable, but the Fornell–Larcker criterion was not met, indicating substantial overlap among the four domains. Accordingly, structural results are interpreted as exploratory rather than as evidence of distinct constructs or causal progression. The reported paths were positive, with managing affect most closely associated with taking action. Student reports clustered around engaging with feedback, using technology-mediated practice, seeking authentic listening exposure, and self-assessing progress. Instructor reports emphasized specific and actionable feedback, structured reflection and goal setting, peer-supported learning, and technology-enabled follow-up. The findings suggest that feedback use in EFL listening involves intertwined cognitive, affective, and behavioral tendencies, but stronger scale validation, longitudinal designs, and direct observation of feedback uptake are required before causal or developmental claims can be made.

## Introduction

Listening is a central component of second- and foreign-language competence because learners must recognize linguistic forms, segment continuous speech, construct meaning, and monitor comprehension under real-time processing constraints (Field, 2008; Goh, 2000; Rost, 2011; Vandergrift and Goh, 2012). English-as-a-foreign-language (EFL) learners frequently report difficulty with rapid speech, reduced forms, unfamiliar accents, lexical gaps, and the integration of bottom-up and top-down information (Field, 2008; Graham, 2006; Goh, 2000; Vandergrift, 2007). These difficulties are not solved simply by increasing exposure. Learners also need information that helps them diagnose where comprehension broke down and select strategies for subsequent listening (Bozorgian and Pillay, 2013; Cross, 2009; Kim, 2017).

Feedback can provide this information, but its presence does not guarantee learning. Feedback may come from instructors, peers, self-assessment, answer explanations, or digital platforms, and students must still interpret its meaning, judge its credibility and relevance, regulate their reactions, and decide what to do next (Boud and Molloy, 2013; Hattie and Timperley, 2007; Nicol, 2010). In listening, this challenge is particularly salient because many comprehension processes are not directly visible. A learner may know that an answer is wrong without knowing whether the problem arose from word recognition, segmentation, inferencing, attention, vocabulary, or background knowledge (Bozorgian and Pillay, 2013; Cross, 2009). Feedback becomes useful only when learners can connect it to a plausible source of difficulty and translate it into targeted practice (Hattie and Timperley, 2007; Pérez-Segura et al., 2022).

This learner-side capacity is commonly discussed as feedback literacy (Boud and Molloy, 2013; Han and Xu, 2020; Nicol, 2010; Poulos and Mahony, 2008). Carless and Boud (2018) define student feedback literacy as the understandings, capacities, and dispositions needed to make sense of feedback and use it to enhance work or learning strategies. Their framework proposes four interrelated features: appreciating feedback (AF), making judgments (MJ), managing affect (MA), and taking action (TA). AF involves recognizing its value and its multiple sources and forms; MJ involves evaluating the quality of one’s own and others’ work; MA concerns responding productively to criticism or disappointing performance; and TA refers to using feedback to change strategies or subsequent work. Together, these features shift attention from feedback as information delivered by a teacher to feedback as a process in which learners actively interpret and use information.

Although feedback literacy has attracted increasing attention in higher education (Fan et al., 2026), several issues remain unresolved in EFL listening. First, language-feedback research has concentrated heavily on writing and visible revision products, whereas listening feedback often targets less observable comprehension processes. Second, the four features are frequently treated as conceptually distinct, but emerging measurement research shows that feedback-literacy dimensions can overlap strongly when assessed through self-report (Dawson et al., 2024; Woitt et al., 2025; Zhan, 2022). Third, research often discusses desirable feedback practices without showing how students and instructors describe the concrete actions that follow listening feedback. A context-specific study can therefore contribute by examining the associations among the four features, while also documenting the practices participants report using to turn feedback into further listening work.

The present study addresses these issues in a large convenience sample of Chinese non-English-major undergraduates. It has two aims: (a) to examine theory-informed structural associations among AF, MJ, MA, and TA in EFL listening, while evaluating the measurement limitations of the four-domain model; and (b) to identify student- and instructor-reported strategies intended to support action on listening feedback. The study does not test the causal effectiveness of these strategies and does not assume that the four features form a fixed developmental sequence.

## A review of the literature

### Feedback as information, dialogue, and learner activity

Feedback has traditionally been defined as information about the gap between current and desired performance (Hattie and Timperley, 2007; Shute, 2008). This information-focused view clarifies why feedback can support error correction and strategy adjustment, but it can also imply that learning follows automatically from the delivery of a sufficiently clear message. Contemporary work instead emphasizes that feedback is a process involving dialogue, sense-making, comparison, and uptake (Boud and Molloy, 2013; Yang and Carless, 2013). The quality of the information remains important, but so do opportunities to ask questions, compare performance with criteria or exemplars, and apply the information in a subsequent task.

Learner-centered feedback models place particular emphasis on agency. Winstone et al. (2017) identify recipience processes through which students seek, process, and use feedback, while Molloy et al. (2020) frame feedback literacy as a set of capabilities that learners can develop in supportive environments. This perspective is relevant to EFL listening because feedback often needs to be converted into a new listening behavior—such as repeated listening, transcript comparison, vocabulary review, attention to reduced forms, or monitoring of inference—rather than into a visible textual revision.

### The four features of feedback literacy

#### Appreciating feedback (AF)

Appreciating feedback is more than expressing a positive attitude. It includes understanding that feedback can improve future learning, recognizing that useful information may come from teachers, peers, self-assessment, or technology, and being willing to revisit feedback over time (Carless and Boud, 2018). In listening, appreciation may encourage learners to treat answer explanations or comprehension breakdowns as diagnostic information rather than as simple scores.

#### Making judgments (MJ)

Making judgments refers to evaluative judgment: the capacity to assess the quality of one’s own or others’ work and to compare performance with criteria or exemplars. In listening, this may involve identifying whether a comprehension error reflects vocabulary, sound discrimination, segmentation, inference, or attention. The capacity is relevant because action depends on a sufficiently accurate diagnosis of what needs to change.

#### Managing affect (MA)

Feedback can evoke embarrassment, frustration, anxiety, defensiveness, or discouragement, especially when repeated listening failures threaten learners’ confidence. Managing affect refers to acknowledging these reactions without allowing them to block interpretation and use of feedback (Carless and Boud, 2018; Liu and Yu, 2022). It does not imply suppressing emotion; rather, it concerns maintaining enough psychological safety and self-regulation to engage with critical information.

#### Taking action (TA)

Taking action is the behavioral expression of feedback literacy (Carless and Boud, 2018). In EFL listening, action may include re-listening with a specific purpose, checking a transcript, practicing difficult sound patterns, reviewing vocabulary, seeking clarification, setting goals, or changing strategy. Because feedback has educational value only when it informs future learning, action is central, but it is also constrained by task design, time, resources, teacher support, and learner motivation.

### Theory-informed associations among the four features

Carless and Boud (2018) describe the four features as interrelated rather than as a fully specified causal model. A theory-informed ordering is nevertheless plausible for empirical examination. Learners who appreciate feedback may be more willing to compare performance and develop evaluative judgment. Appreciation may also support affect management by framing critical information as useful rather than purely threatening. Making more accurate judgments may reduce uncertainty and help learners interpret negative feedback in specific, manageable terms. Finally, learners who manage discouragement or defensiveness may be more likely to act on what they have understood.

These paths should not be read as a universal developmental sequence. Appreciation can coexist with passivity; evaluative judgment can increase anxiety by making weaknesses more visible; and action may occur because of external requirements rather than affective readiness. Reciprocal and alternative relations are possible, but a cross-sectional design cannot establish temporal direction. The present model therefore examines four theory-informed structural associations and interprets them cautiously.

### Feedback literacy in EFL listening and the present contribution

EFL listening research has documented the value of strategy instruction, metacognitive awareness, repeated practice, and technology-supported listening (Jia and Hew, 2022; Vandergrift and Goh, 2012). Personalized feedback can support listening and reading performance, but its value depends on whether learners can identify the relevance of the information and use it in later practice (Pérez-Segura et al., 2022). Feedback-literacy research adds a useful lens because it connects the informational, evaluative, affective, and behavioral aspects of this process.

The present study makes three bounded contributions. Theoretically, it examines whether the four features show the proposed pattern in an EFL-listening context while explicitly testing their empirical distinctness. Empirically, it uses a large student sample but treats convenience sampling and self-report as limitations rather than evidence of national representativeness. Pedagogically, it documents reported student and instructor practices without claiming that these practices were experimentally shown to improve listening.

### Research questions and hypotheses

*RQ1*: What exploratory structural associations are observed among appreciating feedback (AF), making judgments (MJ), managing affect (MA), and taking action (TA) in EFL listening?

*RQ2*: What strategies do Chinese non-English-major university students report using to act on EFL-listening feedback, and what practices do college English instructors report using to encourage such action?

*H1*: AF is positively associated with MJ.

*H2*: AF is positively associated with MA.

*H3*: MJ is positively associated with MA.

*H4*: MA is positively associated with TA.

## Method

### Research design

The study used a quantitatively dominant mixed-methods design. A student questionnaire provided the quantitative strand and included two open-ended prompts. Semi-structured interviews with college English instructors provided contextual qualitative evidence about practices intended to support feedback use. The strands were integrated in the discussion section by comparing the structural pattern with the actions and supports described by participants. Because the instructor and student samples were not designed as matched dyads, instructor accounts are treated as contextual rather than as direct explanations of individual student responses.

### Ethics statement

The study was reviewed and approved by the Research Ethical Review Board of the corresponding author’s institution. Participants received information about the purpose of the study, voluntary participation, confidentiality, and their right to discontinue. Informed consent was obtained before data collection.

### Participants and sampling

A total of 2,079 Chinese non-English-major undergraduates responded to the online survey. The researchers removed 178 records that showed invariant response patterns across the questionnaire, leaving 1,901 cases for analysis. As shown in Table 1, the final sample included 908 men (47.76%) and 993 women (52.24%); 1,273 freshmen (66.96%) and 628 sophomores (33.04%); 95 students who had passed CET-4 (5.00%) and 1,806 who had not (95.00%); and 11 students who had passed CET-6 (0.58%) and 1,890 who had not (99.42%). Recruitment occurred through university-affiliated WeChat groups with support from professors and advisers at 141 universities. This large but convenience-based sample should not be interpreted as nationally representative.

**Table 1:** Student participants’ personal information.

Variable	Category	*N*	%
Gender	Men	908	47.76
	Women	993	52.24
Year	Freshmen	1,273	66.96
	Sophomore	628	33.04
CET4	Passed	95	5.00
	Not passed	1806	95.00
CET6	Passed	11	0.58
	Not passed	1890	99.42
Total		1901	100.00

Eighteen college English instructors were recruited through convenience sampling for semi-structured interviews. Twelve were women and six were men; 13 held graduate degrees and five held undergraduate degrees in EFL-related fields. Twelve had more than 10 years of college EFL-listening teaching experience, and six had less than 10 years.

### Student questionnaire

The questionnaire was developed by the research team to operationalize the four features in Carless and Boud’s (2018) conceptual framework within EFL listening. It should not be described as a scale created or validated by Carless and Boud. The development process began by using the four conceptual features, i.e., appreciating feedback, making judgments, managing affect, and taking action, as the content domains. The research team then generated items that translated each domain into listening-specific situations and behaviors. Items for appreciating feedback addressed the value, sources, forms, and digital accessibility of feedback about listening; making-judgments items addressed evaluation of one’s own and peers’ listening work; managing-affect items addressed reactions to negative listening feedback and feedback-related help seeking; and taking-action items addressed using feedback to guide subsequent listening practice and self-regulation. Generic feedback wording was contextualized by referring explicitly to English listening, listening homework, teacher and peer feedback, digital tools, and follow-up action. The original version contained 20 five-point Likert items (1 = strongly disagree to 5 = strongly agree): five AF items, four MJ items, six MA items, and five TA items. Item TA4 was excluded from the reported measurement model because its loading was below 0.50, leaving 19 indicators (Appendix A). No formal expert review, cognitive interviewing, or pilot study was performed. The questionnaire should therefore be treated as a preliminary, researcher-developed contextual operationalization rather than as an independently validated EFL-listening scale.

The measurement results reported in the results section provide evidence about internal consistency and convergent validity in this sample. They do not, by themselves, establish content validity, cross-cultural validity, criterion validity, or discriminant validity. Recent validated feedback-literacy measures also indicate that rigorous scale development requires explicit construct specification, expert review, piloting or cognitive testing, and independent psychometric evaluation (Dawson et al., 2024; Woitt et al., 2025; Zhan, 2022). The absence of expert review and piloting is acknowledged as a methodological limitation of the present study.

### Student open-ended prompts

Two written prompts were included in the questionnaire (see Appendix B). The purpose of these two open-ended prompts was to elicit actions students reported taking after receiving teacher or peer feedback on English listening.

### Instructor interview guide

The semi-structured interviews were intended to contextualize how instructors attempted to make listening feedback interpretable, emotionally manageable, and actionable. The guide addressed common student reactions to listening feedback, ways of diagnosing listening problems, strategies for supporting reflection and goal setting, uses of peer and technology-mediated feedback, and follow-up activities (see Appendix C). The interviews were not designed to test whether these practices caused improvement in listening performance.

### Data collection

The online questionnaire was distributed through the Survey Star platform via university-affiliated WeChat groups. Participation was voluntary and anonymous or confidential according to the approved protocol. Instructor interviews were conducted after convenience recruitment through professional networks and followed the semi-structured guide reproduced in Appendix C. The interviews elicited instructors’ experiences of student reactions to listening feedback, diagnostic and affective support, reflection and goal setting, peer feedback, technology use, and follow-up activities.

### Quantitative analysis

Quantitative analyses were conducted using SmartPLS 4.0 (Ringle et al., 2022). The measurement model was first evaluated in terms of indicator loadings, Cronbach’s alpha, composite reliability, average variance extracted (AVE), and discriminant validity using the Fornell–Larcker matrix. The structural model was then examined to test H1–H4, with the statistical significance of the path coefficients assessed through bootstrapping with 5,000 resamples. All path estimates were reported as standardized coefficients.

Discriminant validity was interpreted conservatively. Under the Fornell–Larcker criterion (Fornell and Larcker, 1981), discriminant validity is not supported when the square root of a construct’s AVE is lower than its correlation with another construct. The heterotrait–monotrait ratio (HTMT) is a recommended complementary assessment in PLS-SEM (Henseler et al., 2015). However, HTMT values, indicator cross-loadings, rho_A, and additional measurement-model fit indices were not available. They are therefore not reported. This omission limits the completeness of the measurement-model evaluation and does not provide support for discriminant validity. To assess possible common-method influence, full-collinearity VIF values were calculated. Values exceeding 3.3 were treated as a warning of potential common-method bias rather than as conclusive evidence of its presence (Kock, 2015). Given the cross-sectional, self-report design and the concerns regarding construct distinctiveness, the quantitative findings are interpreted as structural associations only. Accordingly, no causal, predictive, mediation, multigroup, or necessary-condition conclusions are drawn.

### Qualitative analysis

Student responses and instructor interviews were analyzed using qualitative content analysis, which is well suited to systematically organizing a large corpus of brief written responses while retaining the meanings conveyed by participants (Creswell and Creswell, 2023; Hsieh and Shannon, 2005). The four members of the research team first color-coded, organized, and sorted responses individually and then collaboratively grouped conceptually similar responses into recurring themes. Collaborative review was used to reconcile different coding decisions, refine code definitions, and agree on the final themes and subthemes. However, no numerical inter-coder reliability statistic, such as Cohen’s kappa or Krippendorff’s alpha, was calculated. The analysis should therefore be understood as a consensus-based team coding process rather than as a formally reliability-tested coding procedure.

Because individual responses could be assigned more than one code, the reported frequencies and percentages for themes are not mutually exclusive and therefore do not necessarily total 100%. The instructor interview data were analyzed using the same general focus on feedback-related action and instructional support. At the same time, the coding process remained open to subthemes that reflected instructors’ distinct experiences, practices, and perspectives.

## Results

### Measurement model

Table 2 reproduces the reported measurement results after removal of TA4. All retained loadings were at or above 0.521. Cronbach’s alpha ranged from 0.764 to 0.834, composite reliability from 0.842 to 0.878, and AVE from 0.520 to 0.621. These results support internal consistency and convergent validity for the retained indicators in this sample, but they do not resolve whether the four domains are empirically distinct. Because HTMT, cross-loadings, rho_A, and additional measurement-model fit indices were not available, the measurement-model evaluation is limited to the indicators reported here and the Fornell–Larcker results below.

**Table 2:** Measurement model analysis.

Construct	Item	Loading	Cronbach’s alpha	CR	AVE
AF	AF1	0.740	0.764	0.842	0.520
	AF2	0.787			
	AF3	0.521			
	AF4	0.777			
	AF5	0.747			
MA	MA1	0.699	0.834	0.878	0.547
	MA2	0.781			
	MA3	0.750			
	MA4	0.758			
	MA5	0.752			
	MA6	0.692			
MJ	MJ1	0.797	0.797	0.868	0.621
	MJ2	0.796			
	MJ3	0.765			
	MJ4	0.794			
TA	TA1	0.792	0.782	0.860	0.605
	TA2	0.741			
	TA3	0.797			
	TA5	0.781			

AF, appreciating feedback; MJ, making judgments; MA, managing affect; TA, taking action; CR, composite reliability; AVE, average variance extracted.

### Discriminant validity and construct overlap

As Table 3 shows, the square root of AVE on the diagonal is lower than several interconstruct correlations. For example, the square root of AVE for AF (0.721) is lower than its correlations with MA (0.800), MJ (0.735), and TA (0.799). The criterion is therefore not met. The four scores show substantial overlap and should not be interpreted as clearly distinct latent constructs on the basis of the reported evidence. HTMT and other complementary discriminant-validity indicators were not available; their absence does not alter the conservative conclusion that discriminant validity was not established.

**Table 3:** Discriminant validity—Fornell–Larcker criterion.

Construct	AF	MA	MJ	TA
AF	0.721			
MA	0.800	0.739		
MJ	0.735	0.801	0.788	
TA	0.799	0.834	0.787	0.778

Diagonals values are square roots of AVE; off-diagonal values are latent-score correlations. The Fornell–Larcker criterion is not met. AF, appreciating feedback; MJ, making judgments; MA, managing affect; TA, taking action.

### Exploratory structural associations (RQ1)

All four theory-informed paths were positive. Table 4 reports the standardized coefficients (*β*). AF was strongly associated with MJ (*β* = 0.735). AF and MJ showed similar unique associations with MA (*β* = 0.459 and 0.463, respectively), and MA was strongly associated with TA (*β* = 0.834). The explained variance was *R*^2^ = 0.540 for MJ, 0.739 for MA, and 0.696 for TA. Because discriminant validity was not established, these coefficients describe an exploratory pattern among strongly overlapping scores rather than a confirmed mechanism among independent constructs.

**Table 4:** Exploratory structural associations and hypothesis testing.

Hypothesis	Path	*β*	t	*p*	*VIF*	*f^2^*	Decision
H1	AF → MJ	0.735	66.506	<0.001	1.00	1.175	Supported, exploratory
H2	AF → MA	0.459	5.339	<0.001	2.18	0.371	Supported, exploratory
H3	MJ → MA	0.463	4.443	<0.001	2.18	0.378	Supported, exploratory
H4	MA → TA	0.834	126.284	<0.001	1.00	2.285	Supported, exploratory

*β*, standardized coefficients; VIF, inner variance inflation factor value; *f*^2^, effect sizes; AF, appreciating feedback; MJ, making judgments; MA, managing affect; TA, taking action.

### Common-method and collinearity diagnostic

The inner VIF for AF and MJ as joint predictors of MA was 2.18, below commonly used thresholds for problematic structural collinearity. However, as shown in Table 5, full-collinearity VIF values were 3.37 for AF, 4.48 for MA, 3.27 for MJ, and 4.26 for TA. Using the conservative 3.3 warning threshold, these values indicate that common-method influence cannot be ruled out. The high interconstruct correlations may therefore reflect both substantive overlap and shared self-report method.

**Table 5:** Full-collinearity VIF values.

Construct	Full-collinearity VIF
AF	3.37
MA	4.48
MJ	3.27
TA	4.26

AF, appreciating feedback; MJ, making judgments; MA, managing affect; TA, taking action.

### Student-reported action strategies (RQ2)

The student responses were organized into four broad themes with subthemes (see Table 6). These categories describe what students reported doing; they do not demonstrate that the practices improved listening proficiency.

**Table 6:** Student-reported action strategies.

Theme	Subthemes	Coded prevalence	Illustrative quotation
Engaging directly with feedback	Recording specific errors; reviewing teacher or peer comments; seeking clarification; revisiting misunderstood vocabulary or pronunciation	*N* = 656	“*Whenever a teacher points out specific pronunciation errors or identifies challenging vocabulary, I make a conscious effort to note these areas for improvement*.”
Technology-mediated listening practice	Using podcasts, recordings, language-learning applications, transcript tools, and comprehension summaries	*N* = 529	“*I frequently engage with English podcasts on topics of personal interest and subsequently evaluate my comprehension by summarizing key points or discussing them with classmates*.”
Seeking authentic listening exposure	Joining English-speaking clubs; watching English-language films or television; attending events; listening to varied accents	*N* = 417	“*By immersing myself in English-speaking environments, I expose myself to various accents, speech patterns, and vocabulary*.”
Self-assessment and action planning	Using checklists; monitoring progress; setting specific goals; selecting targeted exercises	*N* = 321	“*I routinely employ self-assessment checklists to evaluate my listening skills and pinpoint areas where I excel or require improvement*.”

#### Engaging directly with feedback

A total of 656 participants emphasized the importance of engaging directly with feedback during classroom activities, lectures, or language practice sessions. This involves Recording specific errors; reviewing teacher or peer comments; seeking clarification; revisiting misunderstood vocabulary or pronunciation. For example, one first-year student highlighted, “*Whenever a teacher points out specific pronunciation errors or identifies challenging vocabulary, I make a conscious effort to note these areas for improvement*”.

#### Technology-mediated listening practice

A total of 529 participants expressed intentions to use technology to enhance their listening abilities outside of the classroom. They reported using podcasts, recordings, language-learning applications, transcript tools, and comprehension summaries. A sophomore student shared, “*I frequently engage with English podcasts on topics of personal interest and subsequently evaluate my comprehension by summarizing key points or engaging in discussions with classmates*”.

#### Seeking authentic listening exposure

A total of 417 respondents indicated plans to actively seek out authentic listening exposure in real-world settings. This involves joining English-speaking clubs; watching English-language films or television; attending events; and listening to varied accents. Another second-year student elaborated, “*By immersing myself in English-speaking environments, I expose myself to various accents, speech patterns, and vocabulary*”.

#### Self-assessing and action planning

A total 321 of the participants stressed the importance of regularly assessing their own progress in listening comprehension and setting specific improvement goals based on feedback received from instructors and peers. This involves using checklists; monitoring progress; setting specific goals; selecting targeted exercises. For instance, a freshman student shared, “*I routinely employ self-assessment checklists to evaluate my listening skills and pinpoint areas where I excel or require improvement.”* Another sophomore student added, *“I typically devise a personalized action plan targeting areas in need of improvement, such as engaging in listening exercises focused on specific language structures or vocabulary*”.

### Instructor-reported practices for supporting action (RQ2)

Instructor accounts also formed four broad themes. As shown in Table 7, these are reported practices and perceptions, not experimentally verified interventions.

**Table 7:** Student-reported practices for supporting action.

Theme	Subthemes	Coded prevalence	Illustrative quotation
Specific, individualized, actionable feedback	Diagnosing the source of difficulty; linking comments to concrete practice; tailoring suggestions to proficiency	*N* = 18	“*When my students struggle with spoken English because of vocabulary gaps, I recommend tactics such as flashcard practice or online vocabulary resources*.”
Structured reflection and goal setting	Prompting strengths/weaknesses analysis; setting short-term goals; requiring action plans and follow-up	*N* = 14	“*By empowering students to take ownership of their learning, educators can inspire them to pursue listening practice and apply feedback*.”
Peer feedback and collaborative learning	Peer review of oral recordings or comprehension explanations; cooperative diagnosis; peer support	*N* = 9	“*I organize peer feedback sessions where students listen to and provide feedback on each other’s oral recordings or presentations*.”
Technology-enabled practice and follow-up	Interactive listening exercises; audio recordings; quizzes; review of performance data; personalized follow-up	*N* = 7	“*Online platforms can provide interactive listening exercises, audio recordings, and quizzes; teachers can then review performance and offer personalized feedback*.”

#### Specific, individualized, actionable feedback

All instructors emphasized the importance of delivering specific, individualized and actionable feedback tailored to each student’s unique needs and language proficiency level. Rather than merely identifying errors, they advocated for offering constructive feedback that diagnosing the source of difficulty, linking comments to concrete practice, and tailoring suggestions to proficiency. For example, an experienced instructor noted, “*When my students struggle with spoken English because of vocabulary gaps, I recommend tactics such as flashcard practice or online vocabulary resources*”.

#### Structured reflection and goal setting

Fourteen instructors highlighted the significance of promoting reflection and goal setting among students, encouraging them to evaluate their listening performance and pinpoint areas for improvement. This approach entails prompting strengths/weaknesses analysis, setting short-term goals, and requiring action plans and follow-up. Another experienced instructor remarked, “*By empowering students to take ownership of their learning, educators can inspire them to pursue listening practice and apply feedback*”.

#### Peer feedback and collaborative learning

Nine instructors expressed intentions to incorporate peer review of oral recordings or comprehension explanations, cooperative diagnosis, and peer support. An early-career instructor proposed, “*I organize peer feedback sessions where students listen to and provide feedback on each other’s oral recordings or presentations*”.

#### Technology-enabled practice and follow-up

Seven instructors suggested using technology to offer additional avenues for interactive listening exercises, audio recordings, quizzes, review of performance data, and personalized follow-up. Another early-career instructor suggested, “*Online platforms can provide interactive listening exercises, audio recordings, and quizzes; teachers can then review performance and offer personalized feedback*”.

## Discussion

### Measurement interpretation

The most important result is methodological rather than substantive: the four latent constructs did not meet the reported discriminant-validity criterion. This finding is consistent with the conceptual claim that the features of feedback literacy are interrelated, but it also limits any attempt to treat them as independent causes in a sequential model (Dawson et al., 2024; Woitt et al., 2025). The correlations of 0.735–0.834 suggest that respondents who endorsed one positive feedback tendency generally endorsed the others. Such a pattern may reflect a broad feedback-literacy orientation, a higher-order factor, shared wording, social desirability, or common-method variance.

This interpretation aligns with recent efforts to develop and validate feedback-literacy measures. Zhan (2022), Dawson et al. (2024), and Woitt et al. (2025) show that feedback literacy can be operationalized in multiple ways and that conceptual clarity and item-level validation are essential. The present items were contextualized directly from a conceptual framework and include some content that may cross domain boundaries. For example, several MA items concern seeking suggestions and continuous improvement rather than affect alone. This may partly explain why MA correlates so strongly with the other constructs.

### Interpreting the exploratory structural pattern

Within these limitations, the structural pattern is theoretically meaningful. AF was strongly associated with MJ, suggesting that students who recognized the value and availability of feedback also reported stronger evaluative engagement. This is compatible with the feedback-literacy emphasis on students learning to compare, judge, and interpret rather than passively receive comments (Carless and Boud, 2018; Molloy et al., 2020). It should not be interpreted as evidence that appreciation causes evaluative judgment; both may reflect a broader orientation toward active learning.

AF and MJ contributed similarly to MA. One interpretation is that valuing feedback and diagnosing performance may make negative information easier to tolerate. An equally plausible explanation is that confident, self-regulated, or highly motivated students tend to endorse all three tendencies. The cross-sectional design cannot distinguish these accounts.

MA showed the strongest proximal association with TA. This pattern is consistent with research showing that emotions influence whether feedback is approached, avoided, or rejected (Hausman et al., 2023; Liu and Yu, 2022). In EFL listening, repeated failure to recognize speech or understand a passage can threaten confidence, so emotional regulation may be closely linked to persistence. Nevertheless, MA should not be described as a proven “gateway.” The MA subscale also contains help-seeking and improvement items, which overlap behaviorally with action-taking and may inflate the association.

### How the qualitative findings contextualize feedback use

The student themes show that reported feedback use extended beyond reading comments. Students described recording specific weaknesses, using digital resources, seeking authentic exposure, and monitoring their own progress. These practices correspond to the informational, evaluative, and behavioral aspects of feedback literacy. They also reflect established listening pedagogy: repeated exposure, metacognitive monitoring, strategy adjustment, and targeted practice are central to learning how to listen (Vandergrift and Goh, 2012).

Instructor accounts emphasize that feedback literacy is not solely a student responsibility. Specific comments, structured reflection, peer dialogue, and follow-up tasks can make feedback easier to interpret and use. This supports learning-centered and dialogic models in which teacher design creates the conditions for learner agency (Boud and Molloy, 2013; Yang and Carless, 2013). The interviews do not show that these practices were effective, but they identify plausible routines for future intervention studies.

### Alternative explanations

Several alternatives must be considered. General academic motivation, self-efficacy, learner autonomy, and prior success may produce uniformly positive responses across all four domains. Social desirability or acquiescent response style may also elevate correlations because all items are positively keyed self-reports collected at one time. The full-collinearity VIF warning reinforces this concern. Institutional or teacher-level differences may further influence feedback experiences. These alternatives mean that the results cannot establish a distinct psychological sequence from AF to MJ to MA to TA.

### Pedagogical implications

The implications should be treated as design suggestions rather than proven effects. First, instructors can make listening feedback diagnostic and actionable by naming the likely source of a comprehension problem and linking it to a specific follow-up activity. “Listen more” is less actionable than “replay the sentence while marking word boundaries, then compare with the transcript”.

Second, affect-sensitive feedback can normalize error and reduce threat. Teachers can frame listening difficulty as information about a process rather than as a fixed lack of ability, allow private reflection before public discussion, and invite clarification. Brief emotion check-ins or reflective prompts may help students notice defensiveness or discouragement before deciding what action to take.

Third, feedback should be followed by an opportunity to use it. Repeated listening, transcript comparison, revised summaries, goal sheets, and short action plans can close the gap between receiving comments and changing behavior. Peer dialogue and technology can broaden access to examples and practice, but teachers should guide students in judging the credibility and relevance of peer or automated information.

Finally, future interventions should measure actual uptake and listening outcomes rather than relying only on self-report. For example, researchers could code whether students revisit feedback, change strategy, improve performance on parallel tasks, and sustain the behavior over time.

### Limitations

This study has several limitations that must be acknowledged. First, the questionnaire was a researcher-developed contextualization of a conceptual framework rather than an independently validated EFL-listening scale. Although the item wording can be traced to the four conceptual domains and was contextualized to listening, the study did not perform formal expert review, cognitive interviewing, or pilot testing. The reported internal-consistency and AVE results do not compensate for these missing development steps or for the failure of discriminant validity.

Second, all quantitative variables were positively keyed self-reports collected at one time. Common-method variance, social desirability, and general response tendency may have inflated the associations. In addition, complementary measurement-model indicators such as HTMT, cross-loadings, rho_A, and additional fit indices were not available. Consequently, the measurement-model evaluation is incomplete, and no claim of discriminant validity is made.

Third, the design was cross-sectional. It captures students’ current perceptions of feedback in English listening across various universities but does not track changes over time. Additionally, the arrows represent a theory-informed statistical specification, not temporal or causal evidence. Longitudinal, experimental, and cross-lagged research is needed to examine directionality.

Fourth, recruitment through WeChat and convenience networks across 141 universities produced a large but nonprobability sample. The predominance of freshmen and the very small CET-4/CET-6 pass groups limit subgroup inference; accordingly, multigroup analyses were not considered.

Finally, reported strategies were not observed or experimentally tested. They should not be described as effective until future studies connect them to verified feedback uptake and listening outcomes.

## Conclusion

This study examined feedback literacy in EFL listening through four proposed features and through student and instructor accounts of feedback-related action. The four latent constructs were strongly associated, but the reported measurement evidence did not establish that they were empirically distinct. The structural pattern is therefore best understood as an exploratory description of an integrated feedback orientation rather than as a causal sequence. Within that pattern, managing affect was most closely associated with taking action, while qualitative accounts highlighted concrete practices involving feedback review, technology-mediated listening, authentic exposure, self-assessment, clear instructional guidance, reflection, peer learning, and follow-up. The study supports a cautious, learner-centered view of listening feedback: information matters, but so do evaluative judgment, emotional response, opportunity, and action. Stronger measurement and longitudinal evidence are needed to determine how these processes develop and whether particular instructional designs improve listening.

## Data Availability

The raw data supporting the conclusions of this article will be made available by the authors, without undue reservation.
