# Supplementary material for: Feedback literacy in EFL listening: structural associations and reported action strategies among Chinese university students and instructors
Source: Front Psychol. 2026 Aug 5;17:1855025. doi: 10.3389/fpsyg.2026.1855025 (PMC13486148; doi:10.3389/fpsyg.2026.1855025)
Supplement: Supplementary file 1 [file Supplementary_file_1.docx]

**Appendix A**

**Researcher-developed feedback literacy items contextualized to EFL listening**

| Item | Description |
| --- | --- |
| AF1 | I understand and value the role of feedback in improving my English listening. |
| AF2 | I know that feedback about my English listening can come from different sources. |
| AF3 | I use digital tools to access, store, and revisit feedback on my English listening. |
| AF4 | I know that feedback about my English listening can come in different forms. |
| AF5 | I understand and value the role of feedback in becoming an active English listener. |
| MJ1 | I develop the ability to make sound judgments about my peers’ English listening homework. |
| MJ2 | I develop the ability to make sound judgments about my own English listening homework. |
| MJ3 | I participate actively in peer-feedback activities in English listening. |
| MJ4 | I develop evaluative capacity in English listening so that I can make more robust judgments. |
| MA1 | I proactively ask teachers for suggestions about my English listening when needed. |
| MA2 | I develop habits of continuous improvement based on self-feedback in English listening. |
| MA3 | I develop habits of continuous improvement based on teacher and peer feedback in English listening. |
| MA4 | I proactively ask peers for suggestions about my English listening when needed. |
| MA5 | I remain emotionally stable when receiving negative feedback on my English listening. |
| MA6 | I avoid becoming defensive when receiving negative feedback on my English listening. |
| TA1 | I develop strategies for acting on feedback in my English listening. |
| TA2 | I make full use of feedback about my English listening to become a more active listener. |
| TA3 | I draw inferences from feedback about my English listening to improve continuously. |
| *TA4 | I use feedback to self-regulate my English listening. |
| TA5 | I recognize the importance of taking action in response to feedback about my English listening. |

*Note: AF = appreciating feedback; MJ = making judgments; MA = managing affect; TA = taking action. *TA4 was excluded from the reported SEM measurement model because its loading was below .50. Several MA items appear conceptually closer to help-seeking or action and should be reconsidered in future scale development.*

**Appendix B**

**Student open-ended prompts**

Prompt 1: After receiving feedback from your English teacher about your listening, what do you usually do to improve your English listening? Please describe specific actions or strategies.

Prompt 2: After receiving feedback from classmates or peers about your listening, what do you usually do to improve your English listening? Please describe specific actions or strategies.

**Appendix C**

**Instructor semi-structured interview guide**

1. What kinds of difficulties do your non-English-major students commonly experience when receiving feedback on English listening?

2. How do you help students understand the source of a listening-comprehension problem rather than simply know that an answer is incorrect?

3. What do you do when students become discouraged, anxious, embarrassed, or defensive in response to listening feedback?

4. How do you encourage students to reflect on feedback and set specific listening goals?

5. What follow-up tasks or action plans do you use to help students apply feedback in later listening practice?

6. How do you use peer feedback or collaborative activities in listening instruction?

7. How do you use digital platforms, recordings, applications, or quizzes to provide feedback and monitor follow-up?

8. What factors make students more or less likely to act on feedback?

9. Can you describe an example in which a student used feedback productively? What appeared to support that use?

10. Is there anything else instructors should do to help students become more capable users of listening feedback?

**Appendix D**

**Qualitative codebook template**

| Code | Definition | Include | Exclude |
| --- | --- | --- | --- |
| Student—engaging with feedback | Reviewing, recording, clarifying, or revisiting specific feedback information | Mentions noting errors, asking what a comment means, reviewing corrections | General listening practice without reference to feedback |
| Student—technology-mediated practice | Using digital media or tools in response to feedback | Podcasts, apps, recordings, transcripts, online exercises | Technology use unrelated to feedback |
| Student—authentic exposure | Seeking real-world or varied spoken-English input | Clubs, films, events, varied accents | Generic classroom attendance |
| Student—self-assessment/action planning | Monitoring progress, setting goals, or selecting targeted practice | Checklists, goals, action plans, targeted exercises | Vague intention to improve |
| Instructor—specific/actionable feedback | Diagnosing a problem and linking it to a concrete next step | Names source of difficulty and practice task | General praise or criticism |
| Instructor—reflection/goal setting | Structured prompts for self-evaluation and planning | Strengths/weaknesses review, goal sheet, follow-up | Unstructured encouragement |
| Instructor—peer/collaborative support | Using peer dialogue or collaborative diagnosis | Peer review, pair comparison, group discussion | Teacher-only feedback |
| Instructor—technology/follow-up | Digital practice plus monitoring or personalized follow-up | Platform exercises, quizzes, performance review | Technology without feedback or follow-up |
